# Functional Specificity of the Members of the Sos Family of Ras-GEF Activators: Novel Role of Sos2 in Control of Epidermal Stem Cell Homeostasis

**DOI:** 10.3390/cancers13092152

**Published:** 2021-04-29

**Authors:** Fernando C. Baltanás, Cynthia Mucientes-Valdivieso, L. Francisco Lorenzo-Martín, Natalia Fernández-Parejo, Rósula García-Navas, Carmen Segrelles, Nuria Calzada, Rocío Fuentes-Mateos, Jesús M. Paramio, Xosé R. Bustelo, Eugenio Santos

**Affiliations:** 1 Mechanisms of Cancer Program, Centro de Investigación del Cáncer (CIC), Instituto de Biología Molecular y Celular del Cáncer (IBMCC), University of Salamanca-CSIC, E-37007 Salamanca, Spain; mucientescynthia@gmail.com (C.M.-V.); fran_lm@usal.es (L.F.L.-M.); nparejo@usal.es (N.F.-P.); rosula@usal.es (R.G.-N.); nuriacal@usal.es (N.C.); rocfuemat@usal.es (R.F.-M.); xbustelo@usal.es (X.R.B.); 2 Mechanisms of Tumor Progression Program, CIBERONC, University of Salamanca-CSIC, E-37007 Salamanca, Spain; carmen.segrelles@ciemat.es (C.S.); jesusm.paramio@ciemat.es (J.M.P.); 3 Molecular Oncology Division, CIEMAT and Instituto de Investigación Sanitaria Hospital Universitario 12 de Octubre, E-28040 Madrid, Spain

**Keywords:** Sos1, Sos2, GEF, KO, Ras signaling, keratinocytes, skin homeostasis

## Abstract

**Simple Summary:**

The Sos Ras-GEFs are known to participate in a wide range of skin-related diseases including cutaneous cancers, cardio-facio-cutaneous syndromes, or hirsutism. However, the specific functional roles played by the Sos1 and/or Sos2 family members in specific skin compartments remain largely unknown. This report aimed at precisely characterizing the specific functions played by Sos1 and/or Sos2 in keratinocytes, an essential cellular component of the skin. Our data show that Sos1 and Sos2 make overlapping contributions to both keratinocyte proliferation and survival. However, Sos1 seems to have a preferential involvement in regulating the ERK axis, whereas Sos2 seems to control the signaling output from the PI3K axis. We also uncovered an essential role of Sos2 in the control of the population of epidermal stem cells.

**Abstract:**

Prior reports showed the critical requirement of Sos1 for epithelial carcinogenesis, but the specific functionalities of the homologous Sos1 and Sos2 GEFs in skin homeostasis and tumorigenesis remain unclear. Here, we characterize specific mechanistic roles played by Sos1 or Sos2 in primary mouse keratinocytes (a prevalent skin cell lineage) under different experimental conditions. Functional analyses of actively growing primary keratinocytes of relevant genotypes—WT, Sos1-KO, Sos2-KO, and Sos1/2-DKO—revealed a prevalent role of Sos1 regarding transcriptional regulation and control of RAS activation and mechanistic overlapping of Sos1 and Sos2 regarding cell proliferation and survival, with dominant contribution of Sos1 to the RAS-ERK axis and Sos2 to the RAS-PI3K/AKT axis. Sos1/2-DKO keratinocytes could not grow under 3D culture conditions, but single Sos1-KO and Sos2-KO keratinocytes were able to form pseudoepidermis structures that showed disorganized layer structure, reduced proliferation, and increased apoptosis in comparison with WT 3D cultures. Remarkably, analysis of the skin of both newborn and adult Sos2-KO mice uncovered a significant reduction of the population of stem cells located in hair follicles. These data confirm that Sos1 and Sos2 play specific, cell-autonomous functions in primary keratinocytes and reveal a novel, essential role of Sos2 in control of epidermal stem cell homeostasis.

## 1. Introduction

RAS proteins are critical signal transduction regulators that regulate cell proliferation, differentiation, migration, and survival in different cell types [1,2,3]. The Ras GTPases are able to shift between inactive (GDP bound) and active (GTP bound) states in a cycle regulated through their interaction with activating guanine nucleotide exchange factors (GEFs) that facilitate GDP/GTP exchange and deactivating GTPase activating proteins (GAPs) that multiply their intrinsic GTPase activity. Sos1 and Sos2 form the most widely expressed and functionally relevant family of GEFs involved in activation of mammalian RAS proteins by upstream cellular signals [4,5,6,7].

Despite their similar protein structures and cellular expression patterns, a variety of specific functionalities have been reported for Sos1 and Sos2 in different biological contexts [7]. Initial studies showed that Sos1 ablation in constitutive Sos1 knockout (KO) mice causes embryonic lethality [8,9], whereas adult, constitutive Sos2 KO mice are viable and fertile [10]. On the other hand, the development of a conditional tamoxifen-inducible Sos1-null mouse model allowed bypassing the embryonal lethality of Sos1-null mutants and testing of the functional specificity/redundancy of Sos1 and Sos2 in adult animals and tissues [11]. Interestingly, adult Sos1 KO or Sos2 KO mice were perfectly viable but double Sos1/2 DKO animals died very rapidly, suggesting functional redundancy between the Sos1 and Sos2 isoforms at the level of full organismal survival and homeostasis [11]. Nevertheless, a number of reports dealing with functional analysis of different biological systems in these KO animal models support the functional prevalence of Sos1 over Sos2 in physiological processes such as cell proliferation and viability, migration, inflammation, or regulation of intracellular ROS levels [12,13,14]. Likewise, a dominant role of Sos1 has also been described in various pathological processes, including the abnormal constitutive activation of NFkB in most cancer cells [15], the development of BCR-ABL-driven leukemia, and, in particular, skin homeostasis and chemically-induced skin carcinogenesis [16,17,18,19]. On the other hand, more recent reports have also described a hierarchical requirement for Sos2 to mediate cell transformation driven by mutant RAS genes and a differential involvement of Sos2 in EGF-stimulated PI3K/AKT signal transmission [20,21]. It is apparent that additional comprehensive functional studies of specific tissue/cell lineages derived from KO mouse models are needed to fully understand the specific functional roles of Sos1 and Sos2 in different physiological or pathological contexts.

A number of early reports have shown a critical involvement of the EGFR-RAS-RAF signaling in epidermal development and carcinogenesis [22,23,24]. Regarding Sos1/2 contribution to that signaling axis in the skin, it was also shown that expression of a dominant Sos1 transgene caused significant hyperproliferation of the basal keratinocyte compartment, leading to hyperplasia and development of skin papillomas with 100% penetrance, [25,26] and was also able to rescue the skin barrier defect caused by the absence of EGFR [27]. In particular, we showed that the skin of single Sos1 KO mice (but not Sos2 KO mice) presents specific structural alterations, including significantly reduced proliferation rate of epidermal keratinocytes. Interestingly, these defects are markedly worsened in the skin of Sos1/2 DKO mice, which also display an almost complete inability to repair skin wounds. Finally, single Sos1 ablation specifically delayed the onset of chemically-induced skin tumor initiation, decreased tumor growth, and prevented malignant progression of papillomas [16].

We wished to ascertain whether the functional alterations observed in the skin of Sos1- and/or Sos2-deficient mice are cell-autonomous and to further identify specific functional roles played by Sos1 or Sos2 in different cell compartments of the skin. To that end, here we examine 2D and 3D cultures of purified keratinocyte populations isolated from Sos1 KO and/or Sos2 KO mice with an aim at determining whether the defects seen in the epidermis of Sos1/2-deficient mice were keratinocyte-intrinsic or indirectly caused by dysregulation of other cell compartments in the skin and at obtaining further mechanistic clues/insight on the specific signaling contributions of Sos1 and Sos2 in keratinocytes.

Our transcriptomic and functional analyses uncovered a prevalent role of Sos1 regarding transcriptional control and control of overall RAS activation levels in keratinocytes, as well as functionally overlapping contributions of Sos1 and Sos2 regarding control of keratinocyte proliferation and survival and underlying EGFR-Ras signaling pathways, although with a preferential involvement of Sos1 in the Ras-ERK axis and Sos2 in the Ras-PI3K axis. Confirming the functional relevance of Sos2 in epidermal homeostasis, interestingly, our analysis of the skin of newborn mice also uncovered the specific critical requirement of Sos2 for epidermal homeostasis and control of the epidermal stem cell population in newborn and adult animals.

## 2. Materials and Methods

### 2.1. Animal Models

WT, Sos1 KO, Sos2 KO, and Sos1/2 DKO mouse colonies were generated and maintained as described previously [11]. Mice were kept, managed, and sacrificed in the NUCLEUS animal facility of the University of Salamanca according to current European (2007/526/CE) and Spanish (RD 1201/2005 and Law 32/2007) legislation. All experiments were approved by the Bioethics Committee of the Cancer Research Center (#417).

### 2.2. Keratinocyte Isolation and 2D or 3D Cultures

Isolation of primary keratinocytes was performed as described elsewhere [28]. P0–P2 neonates from the four relevant genotypes (WT, Sos1 KO, Sos2 KO, and Sos1/2 DKO) were euthanized and their skin removed and incubated in Keratinocyte-SFM (10725-018, Gibco) containing dispase (250 U/mL, 04942078001, Gibco) and antibiotics (penicillin/streptomycin) for 16 h at 4 °C. Next, the epidermis was mechanically separated from the dermis and placed over 400 μL of accutase (Cnt-Accutase-100, CELLnTEC) for 1 h at 37 °C. The keratinocytes were then mechanically extracted and cultured as indicated.

For 2D cultures, primary keratinocytes (1.5 × 10^6^ cells) were seeded on 10 cm dishes with KSFM medium supplemented with human recombinant EGF, bovine pituitary extract (Gibco and antibiotics (penicillin/streptomycin), and 0.5 M CaCl_2_. Sos1 depletion in Sos1 KO and Sos1/2 DKO primary keratinocytes was achieved by infecting the cells with GFP-Adeno-Cre (Ade5CMVCre-eGFP; VVC-U Iowa-1174; MOI = 2) for 72 h. To avoid off-target effects of the GFP-Adeno-Cre, both WT and Sos2 KO cells were also infected with the adenovirus under the same conditions. Original, primary keratinocyte cultures that had not yet undergone any splitting (passage 0) were used for all experiments involving expression analyses, cell cycle analysis, and studies of actively growing, steady-state keratinocyte cells. Primary keratinocyte cultures in passage 1 were used for the studies of Sos-mediated signaling upon EGF stimulation.

Three-dimensional histotypic cultures were performed as described previously [29]. Briefly, 5 × 10^5^ primary keratinocytes were seeded on 12 mm diameter cell culture plate inserts (PIHP01250 MilliCell, Millipore, Burlington, MA, USA) with KSFM medium supplemented with human recombinant EGF, antibiotics (penicillin/streptomycin), and 0.02 M CaCl_2_ and cultured for 48 h. The medium was then changed to 3D-barrier (CnT-PR-3D, CellnTec, Bern, Switzerland), and the air-lift was performed according to manufacturer’s instructions. The 3D cultures were maintained for 12 days with three changes of medium per week. Sos1 excision was achieved by adding GFP-Adeno-Cre (MOI 1.5) every 4 days. Finally, cells were fixed with PFA (4% *v*/*v*) for 16 h at 4 °C and paraffin-embedded.

### 2.3. Western Blot and Pull-Down Assays

To analyze cell proliferation, induction of apoptosis, and Sos-mediated signaling pathways at steady state, sub-confluent primary keratinocytes of the four relevant genotypes were harvested and protein lysates were obtained using RIPA buffer. To study the effect of Sos1/2 depletion on the regulation of Ras/MAPK and PI3K/AKT signaling pathways upon EGF stimulation, WT, Sos1 KO, Sos2 KO, and Sos1/2 DKO primary keratinocytes were serum-starved for 10 h and then stimulated with EGF (100 ng/mL) at both short-term (2, 5, and 10 min) and long-term (90, 180, 360, and 540 min). Measurement of expression levels of Sos proteins in normal adult skin and neonate skin was performed as follows. P2 (neonates) and P60 (adult) WT mice were sacrificed, and the hair (in adult mice) was removed, and dorsal skin was rapidly isolated and frozen. Posteriorly, the skin was mechanically chopped and homogenized using the GentleMacs^®^ dissociator (Miltenyi Biotec, Pozuelo de Alarcon, Spain) in lysis buffer (9803, Cell Signaling, Danvers, MA, USA) containing 1 mM NaF, 1 mM PMSF, and 1× of Complete^®^ (13539320, Roche Diagnostics GmbH, Mannheim, Germany). Protein concentration was determined using Bradford assay. In all cases, 30 μg total protein was loaded in electrophoresis gels, and immunoblotting was performed as previously described [13].

To determine the levels of the GTP-bound levels of Ras and Rac in primary keratinocytes, we performed pull-down assays for active Ras and Rac as previously reported [13].

Primary antibodies used were: rabbit anti-pAKT^S473^ (#4060, 1:1000, Cell Signaling, Danvers, MA, USA), mouse anti-AKT (#2920, 1:2000, Cell Signaling, Danvers, MA, USA), rabbit anti-cleaved caspase 3 (#9661, 1:1000, Cell Signaling, Danvers, MA, USA), mouse anti-pERK (sc-7383, 1:1000, Santa Cruz Biotechnology, Dallas, TX, USA), rabbit anti-ERK (sc-94, 1:1000, Santa Cruz Biotechnology, Dallas, TX, USA), rabbit anti-PCNA (#13110, 1:1000, Cell Signaling, Danvers, MA, USA), mouse anti-pP70S6K^T389^ (#9206, 1:1000, Cell Signaling, Danvers, MA, USA), rabbit anti P70S6K (#9202, 1:1000, Cell Signaling, Danvers, MA, USA), rabbit anti-pP90RSK^T359/S363^ (#9344, 1:1000, Cell Signaling, Danvers, MA, USA), mouse anti-Ras (005-516, 1:500, Millipore, Burlington, MA, USA), mouse anti-Rac1 antibody (610651; BD, San Jose, CA, USA), mouse anti-Sos1 (610096, 1:500, BD, San Jose, CA, USA), rabbit anti-Sos2 (sc-15358, 1:500, Santa Cruz Biotechnology, Dallas, TX, USA), and mouse anti-tubulin (T5293, 1:10,000, Sigma, St. Louis, MO, USA).

### 2.4. Microarray Analysis

RNA from GFP-Adeno-Cre-treated 2D-cultured primary keratinocytes of the four relevant genotypes (WT, Sos1 KO, Sos2 KO, and Sos1/2 DKO) was extracted with Nyzol and QIAGEN RNeasy Mini kit (74104, QIAGEN) following manufacturer’s recommendations. Purified RNA was hybridized to Affymetrix “Clariom S” mouse array following manufacturer recommendations. All microarray hybridization data were deposited and are available at the NCBI Gene Expression Omnibus (GEO) database. (GSE166020, https://www.ncbi.nlm.nih.gov/geo/query/acc.cgi?acc=GSE166020, accessed on 3 February 2021). R version 3.6.3 was used for statistical analyses, along with Python version 3.9 for text file processing. Signal intensity values were obtained from expression microarray CEL files after robust multichip average (RMA). Differentially expressed genes were identified using linear models for microarray data (Limma). Adjusted *p*-values for multiple comparisons were calculated applying the Benjamini–Hochberg correction (FDR). Gene Ontology and KEGG pathways enrichment analyses were performed using DAVID. Expression heatmaps were generated using the heatmap3 R package.

### 2.5. Cell Cycle Analysis

Subconfluent, GFP-Adeno-Cre-treated (72 h), 2D cultures of primary keratinocytes of the four relevant genotypes (treated in the same conditions than for microarray studies) were harvested and centrifuged for 5 min at 1300 rpm. Supernatant was removed and cells were fixed with 70% ethanol overnight in cold. After washing with PBS, primary keratinocytes were resuspended for 30 min in staining buffer containing 100 μg/mL RNase A, 0.1% Triton X-100, and 50 μg/mL Propidium Iodide in PBS and stored in dark. Fifty thousand events/sample were measured. Data were acquired on an Accuri C6^®^ (BD, San Jose, CA, USA) flow cytometer.

### 2.6. Isolation of Skin Stem Cells

This procedure was performed as previously described [30]. Briefly, the dorsal skin of WT and Sos2 KO (neonatal and adult) mice was excised, cleaned, and digested in 0.25% trypsin (ThermoFisher, 25200056, Waltham, MA, USA) overnight at 4 °C. The cell suspension was then filtered, resuspended in EMEM (Lonza, BE06-174G, Basel, Switzerland), supplemented with 15% fetal bovine serum (ThermoFisher, 10500064, Waltham, MA, USA), incubated for 30 min on ice with biotin-conjugated antibodies to CD34 (1:50, eBioscience, 13-0341-85), and subsequently with both APC-conjugated streptavidin (1:300, BD Biosciences, 554067, San Jose, CA, USA) and PE-conjugated antibodies to CD49f (1:200, MCA699PE) for 30 min. The cell suspension was then incubated with 6-diamidino-2-phenylindole (DAPI) (0.1 ng/μL, Sigma-Aldrich, D9542, St. Louis, MO, USA) for 5 min to detect and exclude dead cells. Finally, CD34/CD49f double-positive cells were isolated and quantified using a FACSAria III flow cytometer (BD Biosciences, San Jose, CA, USA) and analyzed with the FlowJo software (Ashland, OR, USA).

### 2.7. Histological Analysis

P0-P2 neonates from WT and Sos2 KO mice were sacrificed, the skin was isolated and fixed in PFA (4% *v*/*v*) for 24 h, and then paraffin-embedded. Paraffin-embedded skin samples of 3 μm thickness from P0–P2 WT and Sos2 KO mice, or samples from 3D organotypic cultures of the four genotypes (WT, Sos1 KO, Sos2 KO, and Sos1/2 DKO), were cut and stained with hematoxylin/eosin following standard procedures. Other sections were used for both immunohistochemical and immunofluorescence analyses.

For immunohistochemical studies, sections were dewaxed, microwaved in citrate buffer (pH 6), and incubated overnight with anti-Ki67 primary antibody (MAD-000310QD-3, 1:50, Master Diagnostica) at 4 °C. Sections were then incubated with the corresponding biotin-conjugated secondary antibody, followed by Vectastain Elite ABC reagent. The reaction product was visualized by incubating the sections in 0.025% 3.3′-diaminobenzidine and 0.003% H_2_O_2_ in PBS.

For immunofluorescence studies, sections were initially treated as above described. After incubation of primary antibodies, the fluorochrome-conjugated secondary antibodies were applied for 1 h at RT. Primary antibodies used for this technique were: anti-BrdU (1:1000; Accurate Chemical, New York, NJ, USA), anti-filaggrin (PRB-417P-100; 1:500, Covance, Princeton, NJ, USA), anti-loricrin (PRB-145P; 1:500, Covance, Princeton, NJ, USA), anti-keratin K5 (PRB-160P, 1:500, Covance, Princeton, NJ, USA), anti-keratin K10 (MA1-82041, 1:40, Invitrogen, Carlsbad, CA, USA). Late apoptosis was analyzed using in situ cell death fluorescein kit (11684795910, Roche Diagnostics GmbH, Mannheim, Germany) following manufacturer recommendations, and the sections were counterstained with DAPI (1:1000). The percentages of Ki67^+^ and TUNEL^+^ cells were quantified in the same area corresponding to ten different microscopy 20X fields per animal (*n* = 3/genotype) by using Image J software (v 2.0.0).

To analyze proliferation in the hair follicle, the dorsal skin of P60 WT and Sos2 KO mice was shaved. After 72 h, mice were intraperitoneally injected with BrdU (100 μg/g body weight) 1 h before sacrifice. After euthanasia, the skin was collected, fixed in 4% PFA overnight at 4 °C, and paraffin embedded. The number of BrdU^+^ cells per follicle was determined in the same area corresponding to ten different microscopy 40× fields per animal (*n* = 3/genotype) by using Image J software (v 2.0.0).

### 2.8. Label-Retaining Cell Analysis

This procedure was performed as previously reported [31]. Briefly, 10-day-old WT and Sos2 KO mice were intraperitoneally injected with bromodeoxyuridine (BrdU; 20 μL of a 12.5 mg/mL dilution in NaCl 0.9%) every 12 h for a total of 4 injections. Afterward, dorsal skin sections were collected 35 days after the last injection, and BrdU incorporation was measured as the number proliferative cells in the hair follicles. Three different animals of WT and Sos2 KO genotypes were used to count at least 150 follicles.

### 2.9. Statistical Analysis

Statistical analyses were performed with the SPSS v23 software package (SPSS Inc., Chicago, IL, USA) using one-way analysis of variance with Bonferroni tests for parametric data and the Kruskal–Wallis test and Mann–Whitney U test for nonparametric data. Significant differences were considered at *p* value < 0.05.

## 3. Results

### 3.1. Adeno-Cre-Mediated Deletion of Sos1 in Primary Keratinocytes

In previous reports we carried out successful ablation of floxed Sos1 alleles in mice, or cells derived thereof, through the induction of Cre recombinase with tamoxifen or 4-hydroxytamoxifen, respectively [11,13,14,16,17]. However, since tamoxifen appears to affect the viability of keratinocytes in culture even at low concentrations, we decided to test the efficiency of GFP-Adeno-Cre infection as an alternative method to accomplish Sos1 depletion in freshly isolated Sos1 KO and Sos1/2 KO primary keratinocytes. Under the conditions specified in Materials and Methods, we observed that, after 72 h of adenoviral infection, the presence of the GFP-labeled adenovirus was detected in almost all primary keratinocytes in the culture (93%), and Western blot analysis of the GFP-Adeno-Cre-infected keratinocytes (Sos1 KO and Sos1/2 DKO) showed practically complete disappearance of Sos1 protein expression (Figure S1A–C). Thus, for the sake of avoiding any possible off-target effects and facilitating meaningful biological comparisons among cellular samples of all different Sos genotypes, in this report we routinely used this procedure (GFP-Adeno-Cre infection) in all experiments involving Sos1 depletion, and all counterpart cellular samples of the other two genotypes (WT and Sos2 KO) were also treated under the same conditions. We also verified in the cellular samples generated under these conditions that the level of Sos1 protein expression in Sos2-ablated cells was not altered in comparison with WT keratinocytes, and that the levels of Sos2 protein in WT cells were also similar to those observed in Sos1-depleted samples [13].

### 3.2. Specific Transcriptional Alterations of Sos1 KO and/or Sos2 KO Primary Keratinocytes

**Figure 1:**
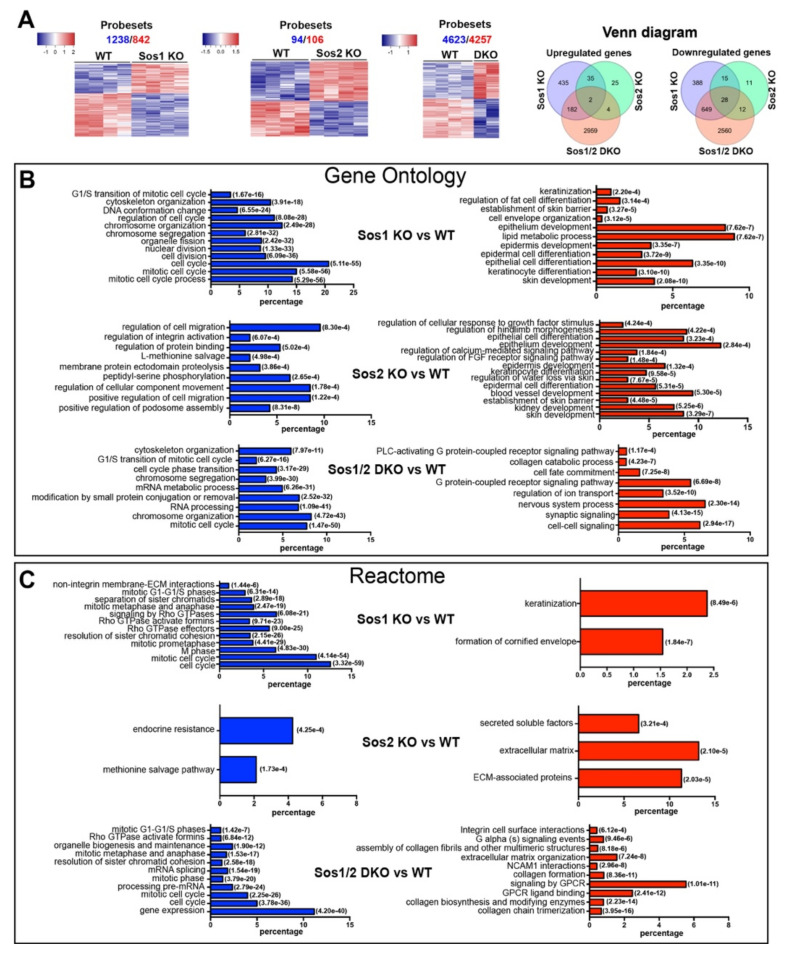
Patterns of differential gene expression in WT and Sos1 KO, Sos2 KO, and Sos1/2 DKO primary keratinocytes. (**A**) Pairwise comparisons of differential gene expression among different SOS genotypes. A set of 14 independent chip microarray hybridizations (4 WT, 4 Sos1 KO, 4 Sos2 KO, and 2 Sos1/2 DKO) were performed using RNA extracted from actively growing primary keratinocytes belonging to the indicated genotypes and analyzed jointly as described in Materials and Methods. The heatmaps show the results of hierarchical clustering and multiclass comparisons identifying the upregulated (red) and downregulated (blue) probesets that showed significant differential expression under highly stringent conditions (FDR = 0.05) when comparing WT primary keratinocytes with the rest of the genotypes, as indicated. Total number of repressed (blue) and upregulated (red) differentially expressed probesets detected in these comparisons is indicated on top of each heatmap. Venn diagrams representing the relations between the lists of differentially expressed genes in the three sets of comparisons. (**B**,**C**) Functional annotation of differentially expressed genes. Text labels on the left side of the bar graphs identify specific functional categories that are enriched at high statistical significance within the lists of differentially expressed genes (red: overexpressed; blue: repressed) included in the clusters identified in each of the 3 indicated pairwise comparisons shown in panel 1A. Each individual bar in the horizontal graph plots represents the percentage of the total number of differentially expressed repressed (blue) or overexpressed (red) gene probesets corresponding to specific groups of genes of each of the above dendrograms that were identified as significantly enriched (hypergeometric *p*-values in italics) for the indicated GO (**B**) and Reactome (**C**) functional categories.

We first used microarray hybridization assays to analyze the impact of Sos1 and/or Sos2 absence on the transcriptional signature of primary keratinocytes (Figure 1). Multiclass comparisons among the lists of differential expression obtained under highly stringent cutoff values (FDR = 0.05) produced dendrograms that clearly discriminated the profile of the WT samples from that of the rest of relevant Sos genotypes (Sos1 KO, Sos2 KO, and Sos1/2 DKO) (Figure 1A). Sos2 depletion had a rather modest transcriptional impact (about 200 differentially expressed probesets relative to WT cells) in comparison with Sos1-depleted cells, which showed 10-fold higher number (~2100) of differentially expressed probesets. Combined depletion of Sos1 and Sos2 showed even higher impact on the transcriptional signature of primary keratinocytes (~8890 differentially expressed probesets), although analysis with Venn diagrams showed that the profile of differentially expressed genes in Sos1/2 DKO cells was significantly more overlapping with that of Sos1 KO than with Sos2 KO keratinocytes (Figure 1A). Whereas the transcriptomic patterns of single Sos1 KO or Sos2 KO cells clearly support a dominant role of Sos1 over Sos2 regarding transcriptional control, the severely aggravated transcriptomic alterations observed in the Sos1/2 DKO cells point to a possible functional redundancy of Sos2 regarding transcriptional control that is only manifested when the other Sos1 isoform is already missing).

Functional annotation analysis of the differentially expressed genes in the dendrograms (Figure 1B) showed that the vast majority of genes downregulated in Sos1-deficient keratinocytes (Sos1 KO and Sos1/2 DKO) are significantly enriched in functional GO categories generally linked with cell proliferation and division, whereas the upregulated genes are mainly ascribed to differentiation and metabolic processes (Figure 1B). In contrast, the much more limited list of differentially expressed genes detected in Sos2 KO cells contains small clusters mostly linked to functional categories of migration and mobility (downregulated genes) or developmental processes (upregulated genes) (Figure 1B). Consistent with the GO annotations, the list of genes differentially expressed in Sos1-deficient cells also showed significant enrichment of downregulated genes clustering to functional categories of the REACTOME database of signaling pathways [32] related to cell cycle, nucleosome assembly, or mitotic processes; of note, a significant downregulation of components of Rho-GTPase-mediated signaling pathways is specifically noted within these categories (Figure 1C). In contrast, the genes upregulated in Sos1-depleted primary keratinocytes showed preferential clustering to pathways of keratinization and formation of the cornified envelope (Figure 1C). Overall, these functional annotations support a significantly prevalent role of Sos1 over Sos2 regarding the transcriptional regulation of proliferations and differentiation processes of primary keratinocytes, and this suggests that Sos1 is required to keep keratinocytes in a proliferating, undifferentiated state.

### 3.3. Functional Overlapping of Sos1 and Sos2 Regarding Control of Cell Proliferation and Survival in Primary Keratinocytes

**Figure 2:**
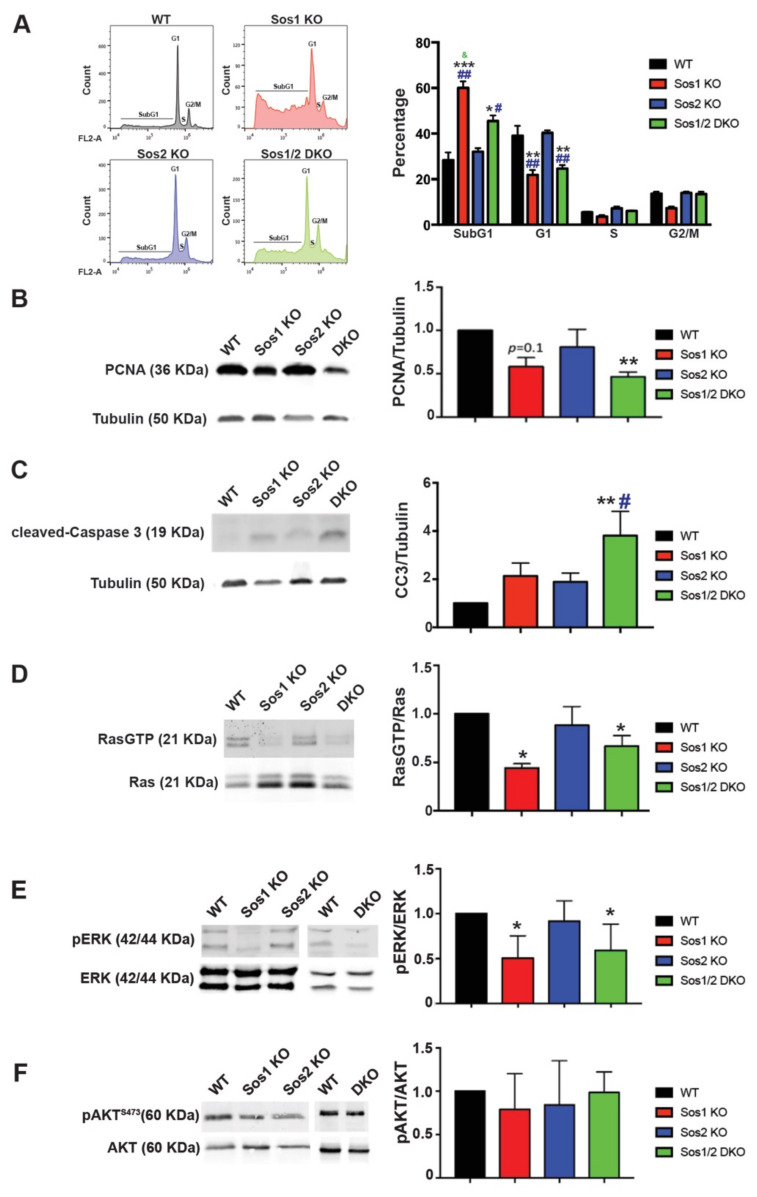
Evaluation of cellular proliferation, survival, and Ras-dependent signaling in primary keratinocytes. We analyzed subconfluent, steady-state cultures of WT (black), Sos1 KO (red), Sos2 KO (blue), and Sos1/2 DKO (green) primary keratinocytes that were actively growing after a period of 72 h infection of the cultures of all genotypes with GFP-Adeno-Cre (MOI 2). (**A**) Cell cycle phase distribution in primary keratinocytes. Left: Representative images of cell cycle profiles of subconfluent primary keratinocyte cultures of the indicated genotypes that were grown in the presence of GFP-Adeno-Cre for 72 h, stained with PI, and analyzed using gated flow cytometry as described in Materials and Methods. Right: Bars represent the percentage of total number of cultured cells corresponding to the indicated phases of the cell cycle. Data expressed as the mean ± SD. * vs. WT; # vs. Sos2 KO; & vs. Sos1/2 DKO. *** *p* < 0.001 vs. WT; ** *p* < 0.01 vs. WT; * *p* < 0.05 vs. WT; ## *p* < 0.01 vs. Sos2 KO; # *p* < 0.05 vs. Sos2 KO; & *p* < 0.05 vs. Sos1/2 DKO. *n* = 3 for each genotype. (**B**) Representative Western blot images illustrating PCNA and tubulin expression in primary keratinocytes of the indicated genotypes. The bar graph depicts the average PCNA/tubulin ratios in each case. *n* = 4 per genotype. ** *p* < 0.01 vs. WT. (**C**) Representative Western blot images displaying cleaved-caspase 3 and tubulin protein expression levels in primary keratinocytes of the indicated genotypes. The bar plot displays the average CC3/tubulin ratios in each genotype. *n* = 4 per genotype. ** *p* < 0.01 vs. WT, # *p* < 0.05 vs. Sos2 KO. (**D**) Ras-GTP pull-down assays performed as described in Materials and Methods on actively growing keratinocytes of the indicated genotypes. Data expressed as normalized ratios between the signals of RasGTP and total-Ras detected in Western immunoblots using a specific pan-Ras antibody. * *p* < 0.05 vs. WT. (**E**) Representative Western blot images showing phosphorylated and total levels of ERK in WT, Sos1 KO, Sos2 KO, and Sos1/2 DKO primary keratinocytes. Graph shows ERK activation levels expressed as normalized ratios between the amount of pERK and total-ERK quantitated by immunoblot assays. * *p* < 0.05 vs. WT. (**F**) Western blot assays represent AKT phosphorylation at steady state in primary keratinocytes of the four genotypes. The graph represents the activation levels expressed as normalized ratios between the amount of pAKT and total AKT. Data expressed as the mean ± SD. (**D**–**F**) *n* = 7/genotype. CC3: cleaved-Caspase 3; PCNA: proliferating cell nuclear antigen.

We next evaluated the impact of Sos1 or Sos2 ablation, individually or in combination, on cell cycle status as well as proliferation and survival of steady-state, actively growing primary keratinocytes of the four relevant Sos genotypes that were maintained in 2D-culture dishes after undergoing similar infection in all cases with adenoCre viruses (to discard off-target effects) (Figure 2).

Consistent with the above microarray data, FACS analysis of propidium iodide (PI)-labeled primary keratinocytes uncovered a decrease of the G0/G1 population in Sos1-deficient (Sos1 KO and Sos1/2 DKO) primary keratinocytes as compared with WT and Sos2-null cells (Figure 2A).

Using PCNA expression level as a marker, we observed that single Sos2-ablation did not alter the proliferation rate of keratinocytes in comparison with WT controls (Figure 2B). In contrast, a slight decrease of proliferation rate was measured in Sos1 KO keratinocytes cultures, and a pronounced reduction of proliferation rate (~2-fold reduction) was always observed in the Sos1/2 DKO (Figure 2B).

Likewise, single ablation of either Sos1 or Sos2 did not result in statistically significant enhancement of the pro-apoptotic cleaved-caspase 3, and only the combined Sos1/2 ablation caused measurable elevation of the expression of this marker of cellular death in comparison with control WT keratinocytes (Figure 2C). However, it should be noted that the percentage of cells in SubG1 (as indicator of apoptosis) was significantly higher in both Sos1-depleted groups (Figure 2A).

Consistent with our previous microarray expression data, these observations suggest functional redundancy of both Sos1 and Sos2 regarding the control of proliferation and cell death/survival of primary keratinocytes in culture, although a partially prevalent role of Sos1 over Sos2 may also be likely, at least in the case of cellular proliferation.

### 3.4. Sos1 Controls Ras Activation Levels in Actively Growing Primary Keratinocytes

Under the same culture conditions, our pull-down assays using GST-Raf RBD fusion proteins showed similar levels of Ras activation (GTP loading) in Sos2 KO primary keratinocytes as in WT cells (Figure 2D). In contrast, RasλGTP levels were significantly lower in Sos1-deficient (Sos1 KO and Sos1/2 DKO) primary keratinocytes (Figure 2D), supporting a critical role for Sos1, but not Sos2, in the maintenance of Ras activation at steady state in growing primary keratinocytes.

Consistent with our prior quantitation of the rates of cellular proliferation and Ras activation, the analysis of downstream Ras-ERK signaling in 2D cultured cells showed that the individual ablation of Sos2 did not alter the levels of extracellular signal regulated kinases 1 and 2 (ERK1/2) phosphorylation in comparison with WT keratinocytes, whereas in contrast, individual or combined ablation of Sos1 in the Sos1 KO or Sos1/2 DKO keratinocytes resulted in significant reduction of the pERK/ERK ratio as compared with WT controls (Figure 2E).

On the other hand, no obvious differences were observed among actively growing primary keratinocytes of the four relevant Sos genotypes regarding activation of downstream phosphatidylinositol 3 kinase (PI3K)/AKT signaling since similar levels of AKT phosphorylation at Ser473 were detected in all cases (Figure 2F).

### 3.5. EGFR Downstream Signaling in Keratinocytes: Differential Involvement of Sos1 in the Ras-ERK Signaling Axis and Sos2 in the Ras-PI3K Signaling Axis

**Figure 3:**
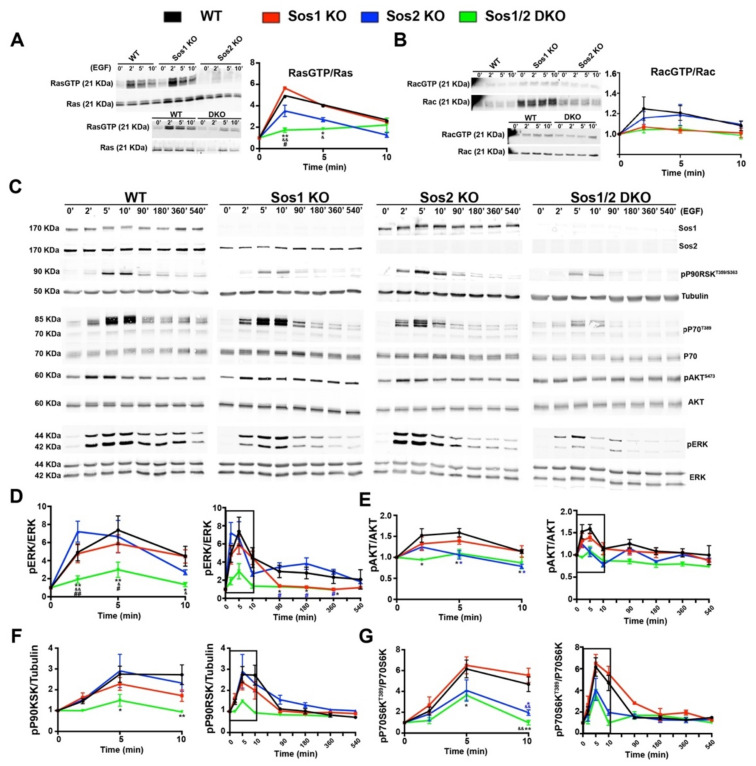
Analysis of short-term and long-term signaling through the EGRF-SOS-RAS axis in primary keratinocytes. Representative Western blot images showing RasGTP/Ras (**A**), RacGTP/Rac (**B**,**C**) Sos1, Sos2, pP90^T359/S363^, tubulin, pP70^T389^, total P70, pAKT^S473^, total AKT, pERK, and total ERK protein expression upon EGF stimulation at the indicated time points in primary keratinocytes of the four defined genotypes. The graphs represent quantitation of ratios of RasGTP/Ras (**A**), RacGTP/Rac (**B**), pERK/ERK (**D**), pAKT^S473^/AKT (**E**), pP90RSK ^T359/S363^/tubulin (**F**), and pP70^T389^/P70 (**G**) ratios at short-term and long-term. Boxed areas in graphs indicate the same data shown in middle panels. Data expressed as the mean ± SD. *n* = 9/genotype. (**A**) * *p* < 0.05 vs. WT; ** *p* < 0.01 vs. WT; & *p* < 0.05 vs. Sos1 KO; && *p* < 0.01 vs. Sos1 KO; # *p* < 0.05 vs. Sos2 KO. (**D**) * *p* < 0.05 vs. WT; ** *p* < 0.01 vs. WT; *p* < 0.05 vs. Sos1 KO; && *p* < 0.01 vs. Sos1 KO; # *p* < 0.05 vs. Sos2 KO; # (in blue) *p* < 0.05 vs. Sos2 KO; ## *p* < 0.01 vs. Sos2 KO. (**E**) * *p* < 0.05 Sos1/2 DKO vs. WT; * (in blue) *p* < 0.05 Sos2 KO vs. WT. (**F**) * *p* < 0.05 vs. WT; ** *p* < 0.01 vs. WT. (**G**) * *p* < 0.05 Sos1/2 DKO vs. WT and Sos1 KO; * (in blue) *p* < 0.05 Sos2 KO vs. WT and Sos1 KO; ** *p* < 0.01 vs. WT and Sos1 KO; && *p* < 0.01 vs. WT and Sos1 KO; && (in blue) *p* < 0.01 Sos2 KO vs. WT and Sos1 KO.

Further evidence supporting specific, distinct functionalities of Sos1 and Sos2 in keratinocytes was provided by our analysis of short-term and long-term signaling through the EGFR-Sos-Ras axis in cells of the four relevant Sos genotypes that had been starved overnight and subsequently stimulated with EGF (Figure 3).

Initial analysis of RasλGTP pull-down assays performed at short times after EGF stimulation showed an almost complete blockade of Ras activation in Sos1/2 DKO keratinocytes devoid of both Sos1 and Sos2, whereas single Sos2-ablated keratinocytes showed a reduced trend of the kinetics of RasλGTP formation in comparison with WT and Sos1 KO cells (Figure 3A). At long-term time points, the RasλGTP signals run close to baseline in all cases and no significant differences were observed among the different genotypes. In addition, no significant differences were detected among all different experimental groups concerning Rac activation upon EGF treatment (Figure 3B).

Consistent with the kinetics of Ras activation, our evaluation of the levels of ERK activation upon EGF stimulation showed that combined Sos1/2 deficiency resulted in almost complete inhibition of EGF-dependent ERK phosphorylation as compared with the rest of the genotypes (Figure 3C), whereas the WT and single Sos1 KO or Sos2 KO cells showed similar levels of ERK activation at short times of stimulation (Figure 3C inset), although Sos2 KO cells appeared to exhibit faster kinetics (maximum reached at 2 min vs. 5 min for the rest of genotypes). A progressive reduction of the level of pERK occurred at longer times of analysis for all genotypes, although the Sos1-deficient cells (Sos1 KO and Sos1/2 DKO) always exhibited statistically significant lower levels of pERK than their WT and Sos2 KO counterparts (Figure 3C,D).

Regarding downstream Ras-PI3K signaling, similar to what happened with the kinetics of ERK activation, the Sos1/2 DKO keratinocytes also showed almost complete blockade of AKT phosphorylation (Ser473) upon EGF stimulation, in contrast with the slight kinetics of activation (pAKT/AKT ratio) observed in the rest of the genotypes (Figure 3C,E). Remarkably in this case (in contrast to what happened with the kinetics profiles of ERK activation in different genotypes), the pAKT profiles kinetics of Sos2 KO cells were very similar to those of the almost completely blocked DKO cells, whereas the Sos1 KO cells showed much more similar kinetics to those of the normal WT control keratinocytes (Figure 3C,E).

The kinetics of EGF-mediated activation of p90RSK, a recognized downstream ERK phosphorylation target [33], were very similar to those of ERK in the different keratinocyte genotypes tested. Thus, Sos1/2 DKO keratinocytes showed almost complete inhibition of p90RSK phosphorylation (T359/S363) (Figure 3C,F), whereas the rest of the genotypes showed similar kinetics of activation profiles, with Sos1 KO keratinocytes exhibiting always lower levels of p90RSK phosphorylation (T359/S363) than their WT and Sos2 KO counterparts (Figure 3C,F).

Likewise, the kinetics profiles of p70S6K [34] phosphorylation (ratio p70S6^Thr389^/total p70S6) upon EGF stimulation were significantly parallel to those of AKT phosphorylation under the same conditions, with Sos2-ablated keratinocytes (both Sos2 KO and Sos1/2 DKO) showing always lower levels of p70S6^Thr389^ than their WT and Sos1 KO counterparts (Figure 3C,G).

Consistent with previous reports [9,20,35], these observations support functional redundancy of Sos1 and Sos2 regarding EGFR-mediated signal transmission, although they also point to a prominent role of Sos2 in regulation of the Ras-PI3K/AKT axis upon EGF stimulation, in agreement with [20,21] and of Sos1 in regulation of the Ras-ERK axis, particularly at long term, in agreement with [9].

### 3.6. Both Sos1 and Sos2 Are Required for Correct Formation of Pseudoepidermis Structures in 3D Keratinocyte Cultures

**Figure 4:**
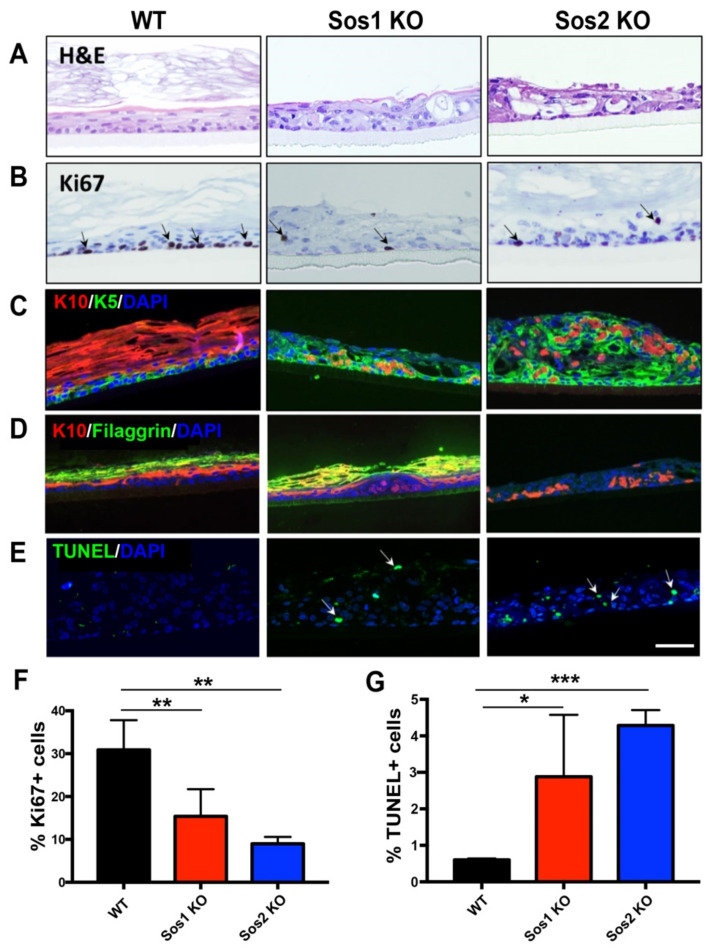
Functional redundancy of Sos1 and Sos2 in the formation of 3D pseudoepidermis. (**A**) Representative H&E-stained sections from histotypic 3D primary keratinocytes cultures of WT, Sos1 KO, and Sos2 KO genotypes. (**B**) Representative images of pseudoepidermis of the indicated genotypes immunostained for Ki67. Arrows show Ki67^+^ cells. (**C**) Representative confocal images of sections of the pseudoepidermis of the three defined genotypes immunostained for K5 (green) and K10 (red). (**D**) Representative confocal images of sections of the pseudoepidermis WT, Sos1 KO, and Sos2 KO genotypes immunostained for K10 (red) and filaggrin (green). (**E**) Confocal images of histotypic 3D cultures stained for TUNEL (green) and counterstained with DAPI (blue). Arrows point out apoptotic cells. Data expressed as the mean ± SD. *n* = 3/genotype. * *p* < 0.05, ** *p* < 0.01 and *** *p* < 0.001 vs. WT. Scale bar: 100 μm. H&E: hematoxylin and eosin; K5: keratin 5; K10: keratin 10. (**F**,**G**) Quantitation of percentage of Ki67 positive (**F**) and TUNEL positive (**G**) cells counted in 3D histotypic cultures of the indicated genotypes.

To try and identify additional functional specificities of Sos1 and Sos2 in keratinocytes, we analyzed the ability of primary keratinocytes isolated from newborn mice of the four relevant genotypes (WT, Sos1 KO, Sos2 KO and Sos1/2 DKO) to grow into histotypic structures recapitulating the normal structure of the epidermis under specific 3D culture conditions [29] (Figure 4).

Our results show that adenoCre-treated primary keratinocytes isolated from our WT mouse strain were able to develop an organized pseudoepidermis, with well-developed and layered cellular stratum and stratum corneum, but Sos1/2 DKO keratinocytes, lacking both Sos1 and Sos2, showed complete abrogation of the ability to grow in 3D cultures (Figure 4A). In contrast, single ablation of either Sos1 or Sos2 allowed the growth of keratinocytes in 3D cultures, but the resulting pseudoepidermis showed significant overall structural disorganization, including defective cellular stratification in the layers of the cellular stratum and alteration of the stratum corneum (Figure 4A).

Analysis of proliferative activity in the WT histotypic cultures showed that the proliferating keratinocytes were mostly located in the basal layer of the cellular stratum, where about 30% of the cell population showed positive immunostaining for the specific marker Ki67 (Figure 4B,F). In contrast, the 3D cultures of individual Sos1 KO or Sos2 KO keratinocytes showed significantly reduced proliferative ability in comparison with WT counterparts, and the Ki67^+^ cells were not restricted to the basal layer of the cellular stratum but were also detected in upper layers (Figure 4B,F), making evident the disorganized structure of the pseudoepidermis that was also previously visualized with H&E staining (Figure 4A).Immunostaining of 3D cultures of the different genotypes using specific markers of keratinocyte differentiation also allowed identification of significant alterations of differentiation in Sos1 KO and Sos2 KO 3D keratinocyte cultures as compared with normal, control WT histotypic cultures (Figure 4C,D). The WT 3D cultures showed very few K5^+^ (immature keratinocytes) cells and a predominant presence of K10^+^ and filaggrin^+^ (mature keratinocytes) in the pseudoepidermis basal layer. In contrast, the patterns of K5 and K10 immunostaining were very significantly altered in both Sos1-ablated and Sos2-ablated 3D keratinocyte cultures, which showed significantly higher numbers of K5^+^ cells, which were also frequently displaced out of the basal layer in more apical layers, as compared with WT cultures. Furthermore, Sos1 KO and Sos2 KO also showed significant reduced numbers of K10^+^ and filaggrin^+^ (mature) keratinocytes in their pseudoepidermal layers, and these more differentiated cells were frequently misplaced to other layers of the pseudoepidermis (Figure 4C,D). These alterations suggest a critical requirement of both Sos1 and Sos2 for correct keratinocyte differentiation and are consistent with the disrupted histological structure detected by H&E (Figure 4) and the transcriptional alterations shown by these genotypes in comparison with WT keratinocytes (Figure 1).

Quantitation of cell death rates in the 3D cultures also uncovered significant differences between WT and Sos1 or Sos2-ablated keratinocytes (Figure 4E,G). Whereas the presence of TUNEL^+^ cells was very limited in WT cultures, a significantly increased percentage (5–10 fold) was observed in the Sos1 KO and Sos2 KO 3D cultures (Figure 4E,G), supporting a relevant role of Sos1 and Sos2 in keratinocyte development.

### 3.7. Specific Alterations of Cell Proliferation, Survival, and Homeostasis in the Skin of Newborn Sos2 KO Mice

**Figure 5:**
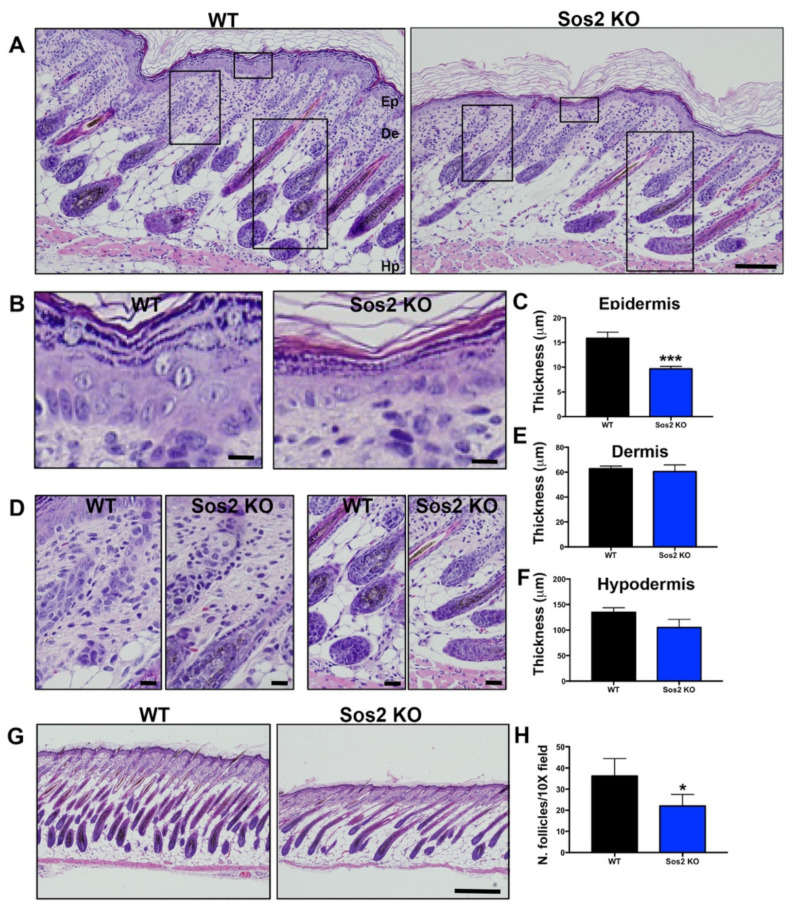
Histological analysis of overall skin architecture in newborn WT and Sos2 KO mice. (**A**) Representative images of H&E-stained, paraffin-embedded sections of skin from newborn WT and Sos2 KO mice. Scale bar: 50 μm. Ep, epidermis; De, dermis; Hp, hipodermis. (**B**,**D**) Higher magnification of boxed areas marked in panel A, focusing on the epidermis (**B**), dermis (left panel in (**D**)), and hypodermis (right panel in (**D**)) of each of the indicated genotypes. Scale bars (**B**,**C**): 10 μm. (**C**,**E**,**F**) Bar plots quantitating thickness of the epidermis (**C**), dermis (**E**), and hypodermis (**F**) layers of the skin of newborn mice of the indicated genotypes. (**G**) Representative images of H&E-stained sections from the dorsal skin of neonate WT and Sos2 KO mice. Scale bar: 200 μm. (**H**) Bar plots represent average of number of follicles per 10× field in the WT and Sos2 KO dorsal skin of neonatal mice. Unfortunately, the embryonal lethality of Sos1 KO mice prevents carrying out similar analyses to evaluate the role of Sos1 in the skin of newborn mice. Data are expressed as means ± the SD. *n* = 4/genotype. * *p* < 0.05 vs. WT; *** *p* < 0.001 vs. WT.

The structural defects shown by 3D histotypic cultures of primary keratinocytes from neonate Sos2 KO mice (Figure 4A) pointed to potential critical role(s) of Sos2 in control of skin homeostasis that could not be detected in our previous studies of the skin of adult Sos2 KO mice [16]. Here we confirmed this notion by documenting the occurrence of a series of developmental alterations in the skin of newborn (P0-P2) Sos2 KO mice (Figure 5). Unfortunately, the embryonal lethality of Sos1 KO mice prevents carrying out similar analyses to evaluate the role of Sos1 in the skin of newborn mice.

In particular, detailed histological comparisons of the overall skin architecture of Sos2 KO and control WT mice confirmed that Sos2 depletion causes specific alterations in the skin histology of newborn mice (Figure 5A). Remarkably, whereas no significant changes were found in the thickness of the dermis or the hypodermis (Figure 5A,D–F), Sos2 single absence resulted in significant reduction of epidermal thickness in newborn mice (Figure 5A–C). In addition, the number of hair follicles was significantly reduced in newborn Sos2 ablated mice as compared with WT mice (Figure 5G,H). It is relevant to mention in this regard that our prior analyses of the skin of adult (2-month-old) Sos2 KO mice did not show any alteration of overall skin histology in comparison with WT mice [16].

**Figure 6:**
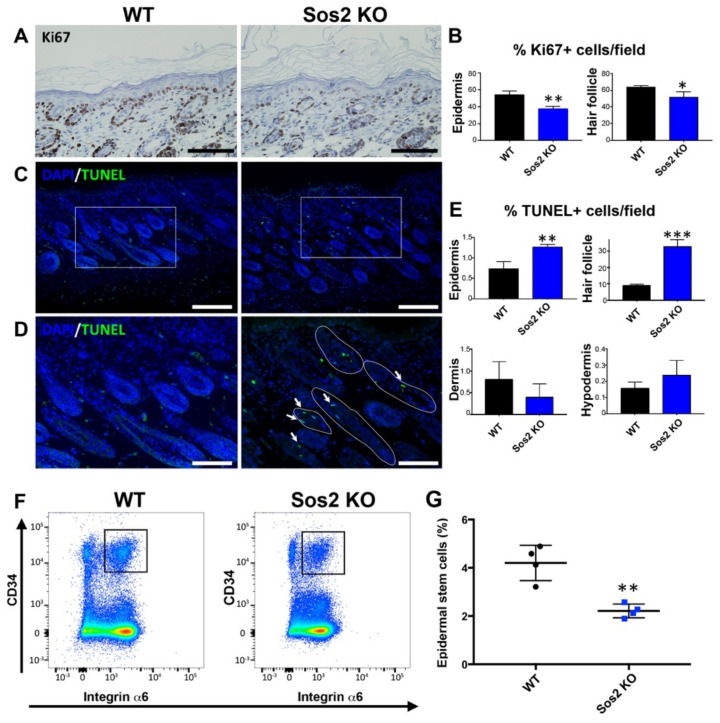
Specific alterations of cellular proliferation, survival, and stem cell population in the skin of newborn Sos2 KO mice. (**A**) Representative images of skin sections of newborn WT and Sos2 KO mice immunostained for Ki67. Scale bars: 50 μm. (**B**) Quantitation of percentage of Ki67^+^ cells per field counted in the epidermis and hair follicles of the indicated Sos genotypes. * *p* < 0.05 vs. WT, ** *p* < 0.01 vs. WT. (**C**) Confocal images of skin sections from animals of the two defined genotypes labeled for TUNEL (green) and counterstained with DAPI (blue). (**D**) Higher magnification of boxed areas marked in panel B, focusing on the hair follicles. White solid lines delimit follicles with TUNEL^+^ cells. Arrows point to apoptotic cells in hair follicles. Scale bars in B: 50 μm, in C: 25 μm. (**E**) Quantitation of percentage of apoptotic TUNEL^+^ cells per field in skin of the indicated Sos genotypes. Data are expressed as means ± the SD. *n* = 3/genotype. ** *p* < 0.01 vs. WT, *** *p* < 0.001 vs. WT. (**F**) Representative flow cytometry scatter plots showing the different epidermal populations according to CD34 and integrin α6 expression in the skin of WT and Sos2 KO neonate mice. The gate used for the isolation of basal bulge epidermal stem cell population is depicted with a square. (**G**) The graph illustrates the quantitation of the abundance of the CD34^+^/integrin α6^+^ pools in the skin of WT and Sos2 KO neonatal mice. ** *p* < 0.01; *n* = 4/genotype.

We next investigated whether the loss of epidermal thickness observed in newborn Sos2 KO mice was a consequence of altered keratinocyte proliferation and/or increased of cell death in this particular skin layer. Specific immunostaining with anti-Ki67 antibodies showed that proliferating cells in the skin of newborn mice are primarily restricted to the basal layer of the epidermis and are also present in the hair follicles (Figure 6A). Of note, quantitative comparison of the immunostained cell populations of WT and Sos2 KO skin showed that single Sos2 ablation significantly reduced the percentage of Ki67^+^ cells, not only in the basal layer of the epidermis but also in the hair follicles (Figure 6A,B).

TUNEL assays detected the presence of apoptotic cells in all layers of the skin of WT or Sos2 KO mice (Figure 6B,C), but quantitative analysis of the percentage of TUNEL^+^ cells found only statistically significant increases in the epidermis, and notably in the hair follicles of newborn Sos2 KO mice as compared with WT counterparts (Figure 6C–E).

We also evaluated whether Sos2 ablation impacted the viability of the population of epidermal stem cells located in the bulge area of the skin hair follicles in newborn mice. Interestingly, our FACS quantitation of specific stem cell markers demonstrated a very significant reduction (about 50%) of the percentage of epidermal stem cells in the skin of newborn Sos2 KO mice as compared with WT counterparts (Figure 6F,G), suggesting a critical role of Sos2 in control of stem cell homeostasis in the skin.

### 3.8. Specific Shrinkage of the Epidermal Stem Cell Population in Hair Follicles of the Skin of Adult Sos2 KO Mice

We reported previously that the overall histological layer structure and the rates of cellular proliferation and survival in the epidermis of adult Sos2 KO mice were not altered in comparison with WT controls [16]. In contrast, our current analyses of the skin of newborn mice as shown in this report (Figure 5G and Figure 6) have clearly shown that Sos2 ablation causes significant defects of homeostasis in the epidermis of newborn mice.

**Figure 7:**
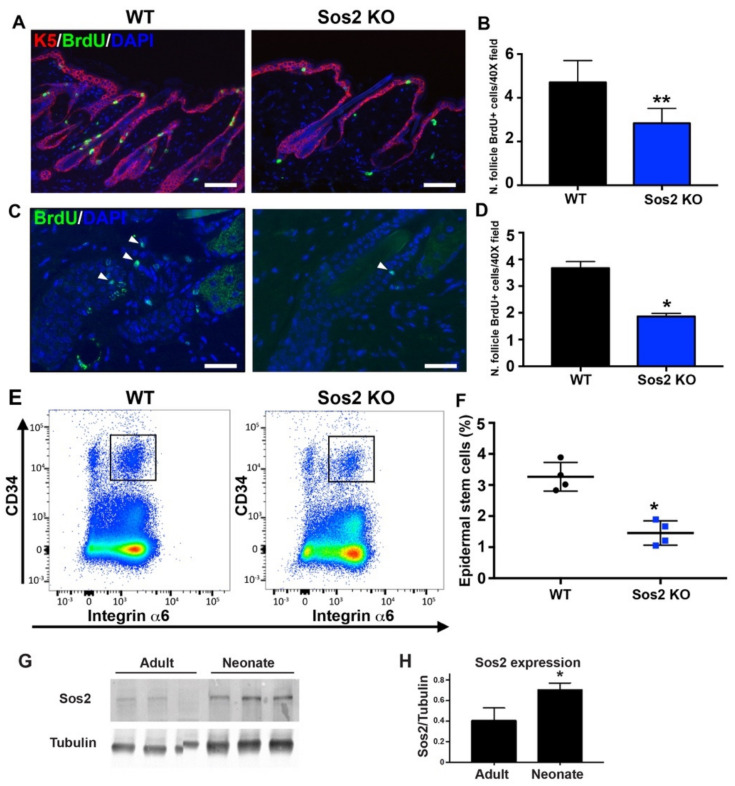
The number and survival of proliferative cell is reduced in hair follicles of the skin of adult Sos2 KO mice. (**A**) Representative confocal images of dorsal skin sections of WT and Sos2 KO adult mice immunolabeled for K5 (red) and BrdU (green) and counterstained with DAPI (blue). Scale bar: 50 μm. (**B**) Graph illustrates quantitation of number of hair follicle BrdU^+^ cells per 40× field. ** *p* < 0.01 vs. WT. (**C**) Representative confocal images of dorsal skin sections of WT and Sos2 KO adult mice from label-retaining experiments (see Materials and Methods) immunolabeled for BrdU (green) and counterstained with DAPI (blue). (**D**) Graph illustrates quantitation of number of survival hair follicle BrdU^+^ cells per 40× field. Arrowheads show proliferative cells. Scale bar: 25 μm. Data are expressed as means ± the SD. *n* = 3/genotype. * *p* < 0.05 vs. WT. (**E**) Representative flow cytometry scatter plots showing the different epidermal populations according to CD34 and integrin α6 expression in the skin of WT and Sos2 KO neonate mice. The gate used for the isolation of basal bulge epidermal stem cell population is depicted with a square. (**F**) The graph illustrates the quantitation of the abundance of the CD34^+^/integrin α6^+^ pools in the skin of WT and Sos2 KO adult mice. * *p* < 0.05 vs. WT; *n* = 4/genotype. (**G**) Western blot analysis shows Sos2 protein expression in dorsal skin from adult (2-month-old) and neonate WT mice. (**H**) Bar plot represent average of Sos2 protein expression in WT dorsal skin of adult and neonate mice. Data expressed as normalized ratios of Sos2 and tubulin protein expression. Data are expressed as means ± the SD. *n* = 3/group. Scale bars: 25 μm. * *p* < 0.05 vs. adult group.

Notwithstanding the fact that the compartment of proliferative cells in the hair follicles was not examined in our initial studies of the skin of adult Sos1/2 KO mice [16], we were interested in determining here whether the negative impact on the epidermal stem cell population detected in Sos2 KO neonates was still maintained during adulthood (Figure 7). Consistent with this notion, our data show that the number of proliferative (BrdU^+^) cells present in the hair follicles of adult Sos2 KO mice was significantly reduced in comparison with adult WT controls (Figure 7A,B). Furthermore, our label-retaining assays show that the rate of survival of proliferative cells in the hair follicle was also significantly reduced in the skin of adult Sos2 KO mice as compared with WT mice (Figure 7C,D). Consistently, FACS quantitation of the percentage of CD34^+^/Integrinα6^+^ stem cells present in the skin of adult animals confirmed that the population of stem cells in the skin of adult Sos2 KO mice was not recovered after birth and continued to be significantly lower (about 50%) than in normal WT controls (Figure 7E,F). The notion of a previously unrecognized functional role of Sos2 regarding maintenance of epidermal skin homeostasis, in particular during the neonatal period, is also consistent with our detection by WB of significantly higher levels (almost 2-fold) of Sos2 protein expression in dorsal skin samples from neonates than in corresponding skin samples of WT adult mice (Figure 7G,H). Furthermore, significantly higher levels of Sos2 mRNA expression than Sos1 mRNA expression were consistently detected in our analyses of actively growing keratinocytes (Figure S2A) or the population of skin stem cells during mouse lifetime (Figure S2B).

## 4. Discussion

Previous reports using specific cell lines and genetically modified mouse models support a critical requirement of Ras-mediated signaling, including the specific contribution of Sos1/2 RasGEF activators, for skin development, homeostasis, and tumorigenesis [16,22,23,24,25,26]. Here, we focused on analyzing primary keratinocytes isolated from the skin of our Sos1/2 KO mouse strains [11] in an effort to unravel the specificity or redundancy of the functional roles played by Sos1 or Sos2 in this essential cellular compartment of the skin. Our results reveal the existence of overlapping but distinct functional contributions of Sos1 and Sos2 to the regulation of signaling pathways controlling proliferation or differentiation of primary keratinocytes, and also uncover an essential, specific role of Sos2 in control of the epidermal stem cell population in mice.

Our initial microarray hybridization analyses of the RNAs from actively growing keratinocytes devoid of Sos1 and/or Sos2 uncovered a highly dominant role of Sos1, and a much more limited role of Sos2, regarding the control of the transcriptional profile of mouse keratinocytes, although concomitant ablation of both Sos1 and Sos2 produced markedly higher alterations of the transcriptional profile than single Sos1 ablation. In any event, our functional annotation analyses uncovered significant enrichment of functional categories related to cell proliferation in the lists of differential gene expression of Sos1-ablated keratinocytes (both Sos1 KO and Sos1/2 DKO) than in WT and Sos2 KO samples, supporting a highly dominant role of Sos1 over Sos2 in control of keratinocyte proliferation. Consistent with the transcriptomic analyses, direct analysis of primary keratinocytes growing on culture dishes also showed significant alteration of cell cycle and reduction of proliferative rates in Sos1-ablated keratinocyte cultures as compared with the unchanged rates of WT or Sos2 KO cultures. Overall, these data support a dominant role of Sos1 over Sos2 in control of keratinocyte proliferation and are consistent with previous reports analyzing the functional impact on various skin cellular compartments of exogenously expressed Sos1 transgenes [25,27] or Sos1/2 null mutations [7,11,13,16]. The notion of a critical role played by Sos1 in keratinocyte proliferation is also supported by a recent report showing that miR-181a, known to specifically target the Sos1 gene, inhibits proliferation in primary keratinocytes [36].

The prominent role of Sos1 in keratinocyte proliferation supported by our transcriptomic analysis was also confirmed with direct measurements of Ras-dependent signals in actively growing keratinocyte cultures of the four relevant Sos genotypes. Indeed, we could only detect significant inhibition of the steady state levels of activated Ras and ERK signaling molecules (RasλGTP and pERK) in the cultures of Sos1-ablated (Sos1 KO and Sos1/2 DKO) cultures, as opposed to the WT and Sos2 KO cultures growing under the same conditions. These observations are also consistent with our previous detection of specifically decreased levels of pERK in immunostained sections of the dorsal skin of Sos1-ablated mice [16] that pointed to a major contribution of Sos1 to the control of in vivo skin cellular proliferation [7,37].

Analyses of short-term and long-term signaling after EGF stimulation of cultured keratinocytes of the four relevant Sos genotypes added further insights to our understanding of the functional roles played by Sos1 and Sos2 in keratinocyte signaling. In particular, these studies showed that both Sos1 and Sos2 are overlapping contributors to EGFR downstream signaling but also clearly demonstrated a predominant role of Sos1 in control of the downstream Ras-ERK axis and of Sos2 in control of the Ras-PI3K axis. The data on the Ras-ERK signaling axis in keratinocytes confirm, and also extend with regard to Sos2, our previous immunological and biochemical studies of whole sections of dorsal skin or isolated fibroblasts from our Sos KO mouse strains that provided experimental support for the essential role of (only) Sos1 in control of skin cell proliferation or in long-term activation of the Ras-MAPK pathway [9,13,16]. On the other hand, this current demonstration of the functional relevance of Sos2 for Ras-PI3K signals in keratinocytes is also consistent with recent reports showing hierarchical requirement for Sos2 to mediate cell transformation driven by mutant RAS genes and a differential involvement of Sos2 in EGF-stimulated PI3K/AKT signal transmission [20,21].

Regarding the control of cell death and survival, Sos1 ablation caused reproducible rates of apoptosis in cultures of primary keratinocytes, suggesting a contribution of Sos1 to modulation of the cellular processes controlling keratinocyte survival. These observations confirm our previous studies of the skin of adult Sos1-depleted mice [16] and could presumably also be directly related to the significant reduction of Ras activation observed in Sos1-ablated keratinocytes.

In contrast with the observations regarding Ras-ERK activation, although Rac-mediated signaling has been reported to play significant roles in skin homeostasis [38,39,40,41,42], we did not detect any differences of RacλGTP levels among the different keratinocyte genotypes tested in our assays. These observations in mice keratinocytes are consistent with the previous immunohistochemical characterization of the dorsal skin of Sos1/2-ablated mice [42] and a report showing the dispensability of Sos1 for E-cadherin-induced Rac1 activation in human foreskin keratinocytes [43]; the observations also support the notion that other Rac-GEFs, rather than Sos, [28] may be responsible for Rac activation in keratinocytes.

A major advantage of 3D keratinocytes cultures over the traditional 2D monolayer cultures is the possibility of trying to recapitulate the in vivo three-dimensional stratified structures housing the keratinocytes in the skin. Despite the apparent inconsistency with previous studies in 2D cultures, our 3D studies have generated complementary and useful information on keratinocytes, having allowed identification of a novel functional role of Sos2 that was not detectable in prior 2D studies of this cell type. In this regard, we observed that whereas combined ablation of Sos1 and Sos2 completely abrogated the ability to grow under 3D culture conditions, the single Sos1 KO or Sos2 KO keratinocytes were able to grow as 3D pseudoepidermis cultures, but these structures displayed a highly disorganized layer structure associated also with reduced cell proliferation, altered maturation, and increased apoptosis of the keratinocytes in comparison with normal WT histotypic cultures. These observations support the functional redundancy of Sos1 and Sos2 regarding proliferation, survival, and spatial organization of skin keratinocytes in vivo and, in particular, uncover a novel, previously unrecognized functional role of Sos2 in control of keratinocyte proliferation and maturation that was previously attributed only to Sos1 since Sos2 depletion did not alter the overall histological structure of the skin in adult mice [16]. Interestingly, differential behaviors displayed by Sos-KO cells growing under 2D or 3D culture conditions have also been described in some recent reports [35,44].

To try and understand the critical role of Sos2 in keratinocyte proliferation and survival that was uncovered here in 3D cultures but went unrecognized in previous studies of the dorsal skin of adult, 2-month-old KO mice [16], we speculated that the visibility of this novel Sos2 functionality could be more strongly manifested during early stages of skin development. Consistent with this notion, our analysis of the skin of newborn (P0/P2) WT and Sos2 KO mice detected a significant reduction of epidermis thickness and total number of hair follicles, together with a reduced rate of proliferation and increased rate of cell death in the hair follicles of the skin of newborn Sos2 KO mice, as compared with WT controls. Most remarkably, the skin of newborn Sos2 KO mice showed significant reduction of the population of epidermal stem cells located in the bulge of the hair follicles. Interestingly, similar comparisons of the population of hair follicles in the skin of adult (2-month-old) WT and Sos2 KO mice (not tested in our previous analysis [16]) showed that the original defect of the stem cell population detected in newborn Sos2 KO mice was maintained without correction during adulthood. It is now apparent that our failure to recognize the functional relevance of Sos2 in skin cell proliferation in previous studies of the skin of adult mice was due to not having tested the hair follicle cellular compartment in those studies [16].

To date, scarce information is available regarding potential functional role(s) of Sos1 and/or Sos2 in human skin. In this regard, gain-of-function mutations of Sos1/2 have been reported in RASopathies, particularly in patients with Noonan syndrome, who may develop skin abnormalities as well as granular skin tumors [7]. In addition, fibroblasts isolated from the skin of patients with hirsutism show increased levels of Sos1 expression [45]. Moreover, upregulated levels of circulating RNA for Sos1 have been detected in the plasma of melanoma patients [7], and a recent report has also demonstrated that enhanced expression of Sos1 is predictive of poor prognosis in uveal malignant melanoma patients [7]. Finally, genetically mediated abrogation of Sos1 in human melanoma cell lines strongly reduces the migratory ability of these tumor cells [7].

Altogether, the data in this report demonstrate the existence of relevant, differential functional roles played by Sos1 or Sos2 in the regulation of keratinocyte proliferation and a much lower relevance, if any, of these two Ras-GEF isoforms regarding control of keratinocyte survival. Importantly, our observations also uncovered a previously unrecognized [7,11,13,16], critical role of Sos2 in control of keratinocyte proliferation and survival during the early stages of skin development in neonates and a similar critical functional role in control of the epidermal stem cell population located in the hair follicles, not only at early stages of development but also during adulthood. Since intercellular crosstalk between epidermal keratinocytes and dermal fibroblasts is critical for the maintenance of skin homeostasis [46], it could be argued that dermal fibroblasts (also devoid of Sos2) in Sos2 KO mice might exert some functional influence over epidermal keratinocytes. However, the fact that the dermal layer is not altered in neonatal Sos2 KO mice, and that our previous work demonstrated the dispensability of Sos2 for regulation of fibroblast proliferation or migration and wound healing in the skin [13,16], suggest otherwise and favor a direct, cell-autonomous functional role of Sos2 in the population of skin keratinocytes as responsible for the defective phenotypes associated with Sos2 ablation that have been described in this report. We also speculate that the differential effects caused by Sos1 or Sos2 ablation in keratinocytes could be due not only to intrinsic mechanistic differences between the two Sos isoforms but also, at least in part, to their different levels of expression in this particular cell type of the skin. Indeed, we observed significantly higher levels of Sos2 RNA than Sos1 RNA in our transcriptomic analyses of actively growing keratinocyte cultures and during different developmental stages of mouse epidermal stem cells [30]. Importantly, an essential, cell-autonomous role of Sos2 in control of the homeostasis of skin keratinocytes also identifies Sos2 as a novel, potential therapy target (besides Sos1) for prevention and/or treatment of epidermal tumors.

## 5. Conclusions

In this report, we identify previously undefined, specific functions played by the members of the Sos family of RasGEFs in primary keratinocytes. Our results reveal that Sos1 and Sos2 make overlapping contributions to keratinocyte proliferation and survival, with Sos1 having predominant involvement in the ERK axis and Sos2 in the PI3K axis of Ras signaling pathways. Importantly, we unveiled a novel, essential role of Sos2 in control of the population of epidermal stem cells that are essential for replacing, restoring, and regenerating the mouse epidermis. These observations support the notion of Sos2 as a potential new therapy target for prevention and/or treatment of epidermal tumors.

## Data Availability

All microarray hybridization data were deposited and are available at the NCBI Gene Expression Omnibus database (GSE166020, https://www.ncbi.nlm.nih.gov/geo/query/acc.cgi?acc=GSE166020 accessed on 28 April 2021). The additional data presented in this study are available on request from the corresponding author.

## Supplementary Materials

The following are available online at https://www.mdpi.com/article/10.3390/cancers13092152/s1, Figure S1: Adeno-Cre mediated ablation of Sos1 in primary mouse keratinocytes. Figure S2: Comparison of Sos1 and Sos2 expression levels in different skin cell compartments.
