# Supplementary material for: Functional Specificity of the Members of the Sos Family of Ras-GEF Activators: Novel Role of Sos2 in Control of Epidermal Stem Cell Homeostasis
Source: Cancers (Basel). 2021 Apr 29;13(9):2152. doi: 10.3390/cancers13092152 (PMC8124217; doi:10.3390/cancers13092152)
Supplement: Supplementary file 1 [file cancers-13-02152-s001.zip › supp + wb/Original blots.pdf]

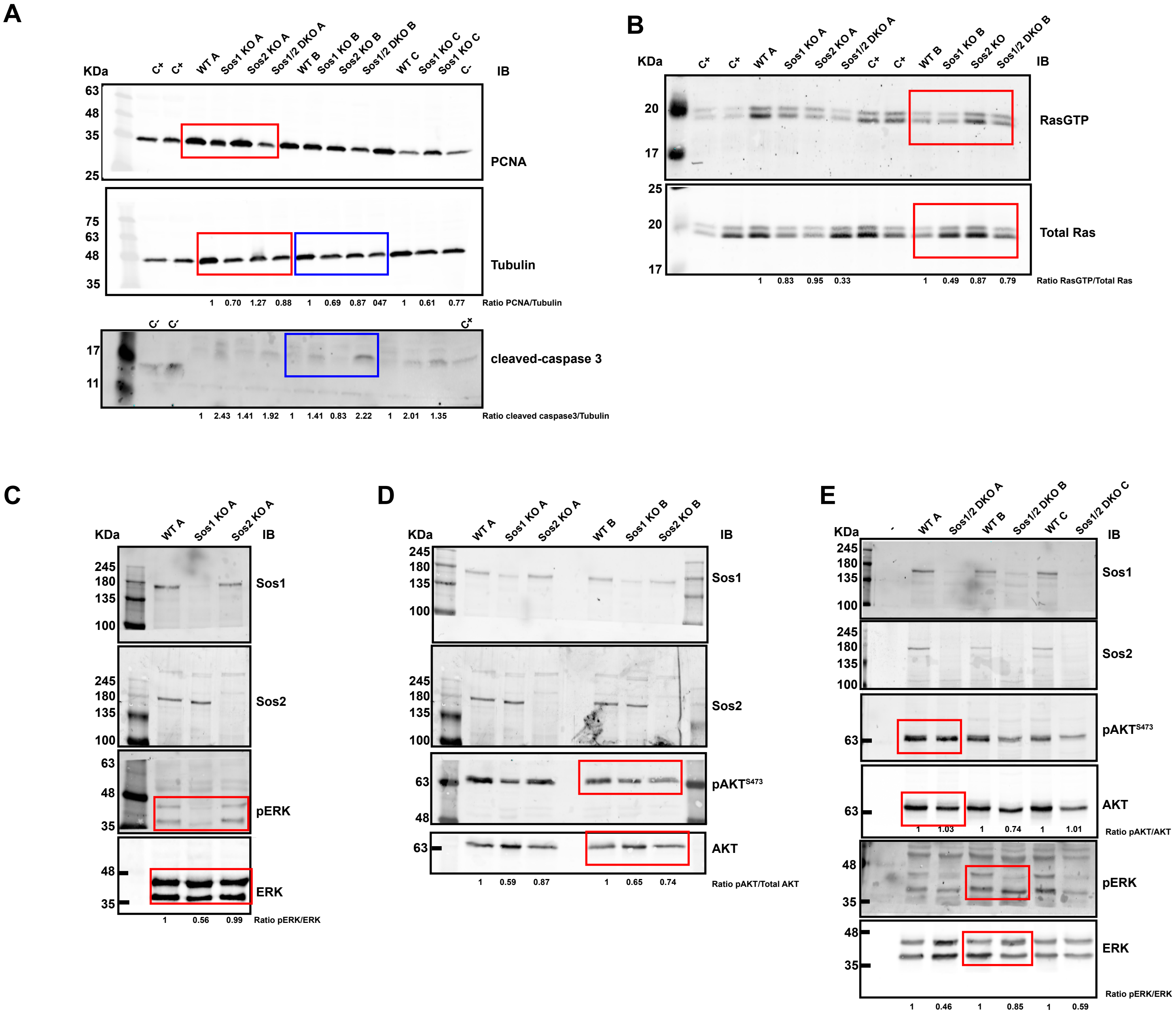

Original plot 1. These plots contain the images showed in Figure 2 of the main manuscript. In *upper panel A*, C+ represent positive control samples of mouse embrionyc fibroblasts proliferation (also used in B to distinghish Ras activation) whereas C- represent a sample with impaired proliferation. On the other hand, these samples also serve as negative and postive control of apoptosis, respectively (lower panel in A). Boxes (red and blue) frame the lines shown in the main text.

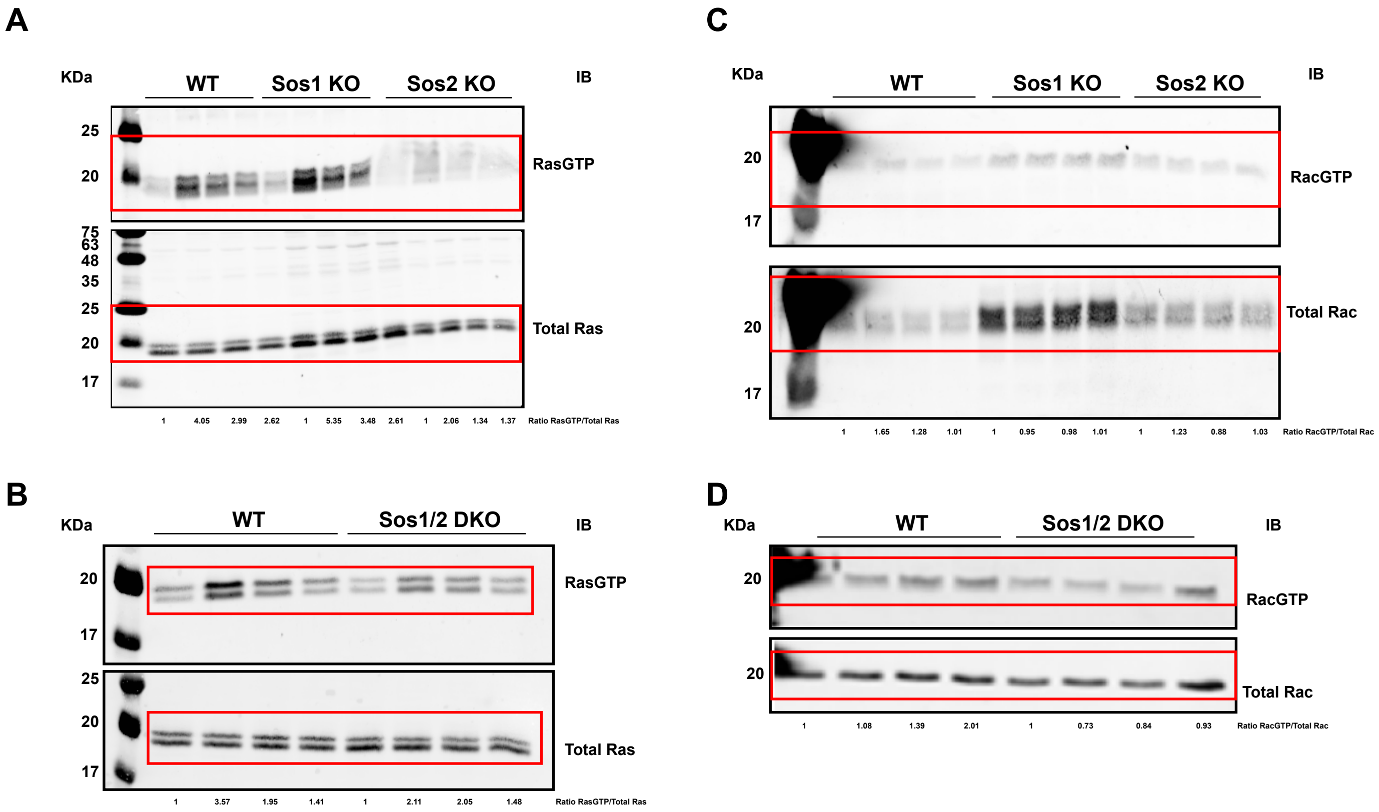

Original plot 2. These plots contain the images showed in Figure 3 A and B of the main manuscript. Red boxes frame lines shown in the main text.

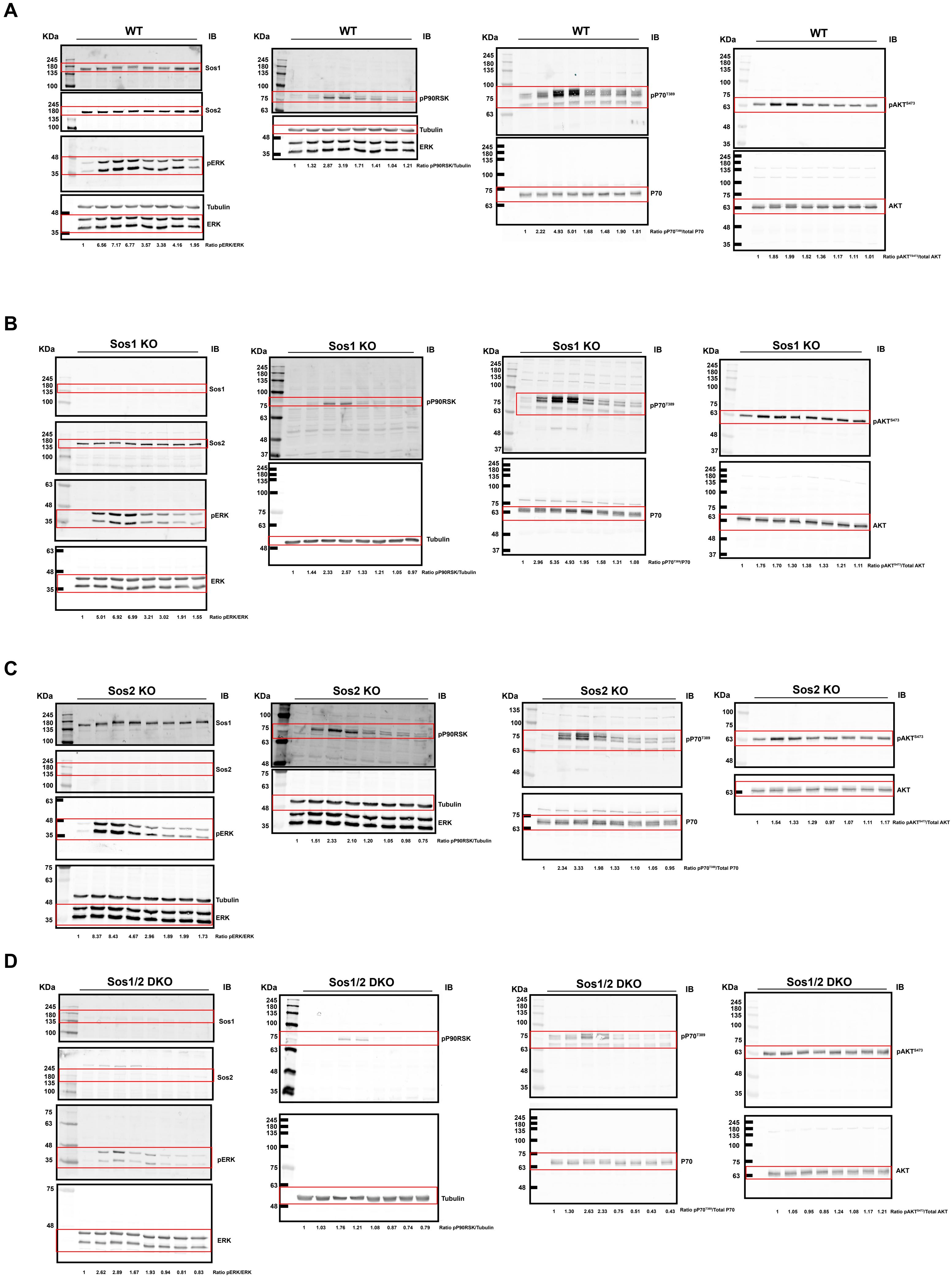

Original plot 3. These plots contain the images showed in Figure 3C of the main manuscript. Boxes frame the lines shown in the main text.

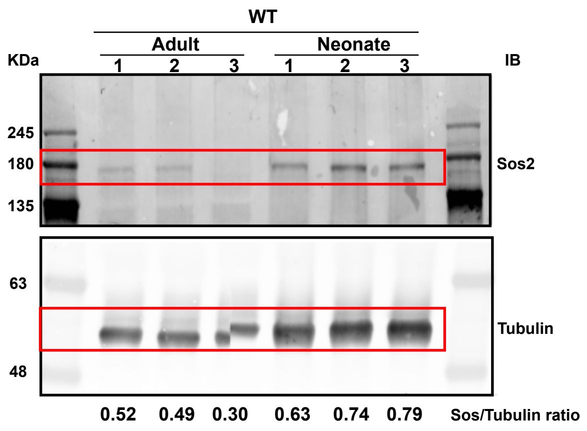

Original plot 4. These plots contain the images showed in Figure 7G of the manuscript. Red boxes frame lines shown in the main text.
