# Supplementary figures and images for: Functional Specificity of the Members of the Sos Family of Ras-GEF Activators: Novel Role of Sos2 in Control of Epidermal Stem Cell Homeostasis
Source: Cancers (Basel). 2021 Apr 29;13(9):2152. doi: 10.3390/cancers13092152 (PMC8124217; doi:10.3390/cancers13092152)

**A**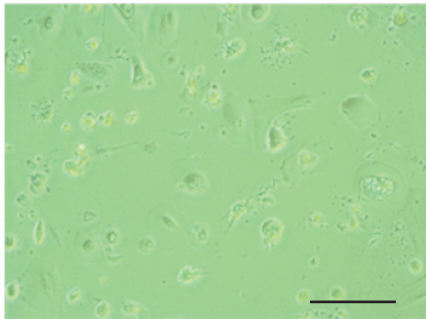**B**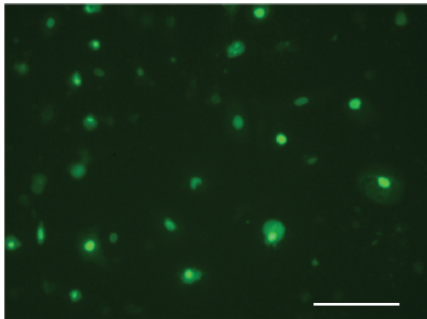**C**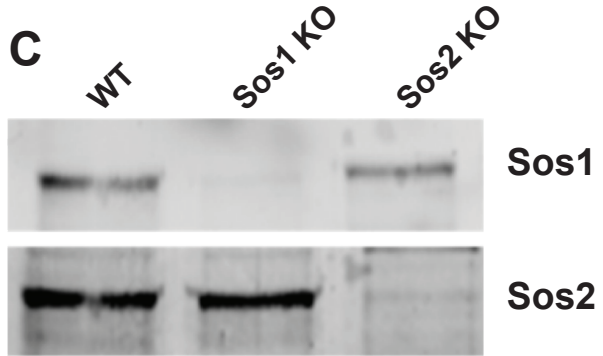

Supplement: Supplementary file 1 [file cancers-13-02152-s001.zip › supp + wb/Supplementary Figure 1.pdf]

**A**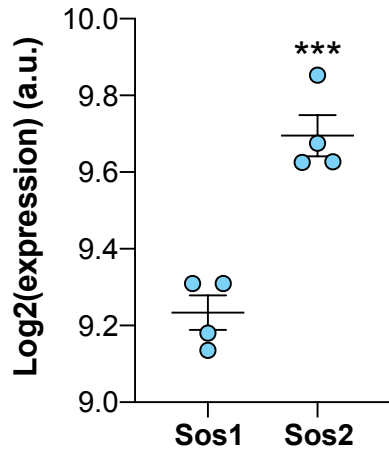**B**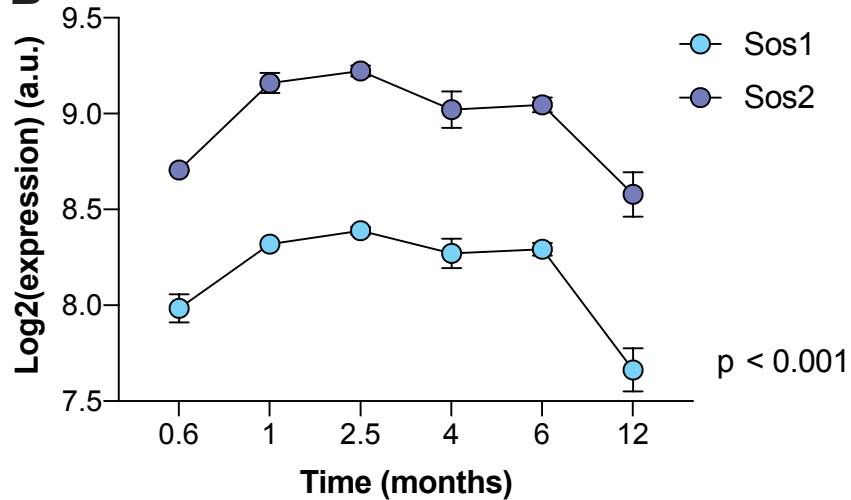

Supplement: Supplementary file 1 [file cancers-13-02152-s001.zip › supp + wb/Supplementary Figure 2.pdf]
